# A Functional SNP in the AMH Gene Is Associated with Litter Size in Dazu Black Goats

**DOI:** 10.3390/ani16121829

**Published:** 2026-06-14

**Authors:** Lei Wang, Yiyu Zhang, Qiuyan Li, Yingping Xia, Cong Zhou, Ying Gong, Xinyang Yao, Xinyan Zhu, Zhen Wang, Ke Zhang, Xiaowei Sun, Dejun Xu, Zhongquan Zhao

**Affiliations:** 1 Chongqing Key Laboratory of Herbivore Science, College of Animal Science and Technology, Southwest University, Chongqing 400715, China; wanglei19970117@163.com (L.W.); y13022356614@163.com (Y.Z.); 17836156708@163.com (Q.L.); 18828815917@163.com (Y.X.); 13769556787@163.com (C.Z.); 18769908822@163.com (Y.G.); 15713939242@163.com (X.Y.); 13543729062@163.com (X.Z.); 2 Chongqing Animal Husbandry Technology Extension Station, Chongqing 401121, China; wzhen2004@163.com (Z.W.); zk8134@163.com (K.Z.); 3 Yunnan Animal Husbandry Station, Kunming 650224, China; sxwxmzz@163.com

**Keywords:** *Capra hircus*, fecundity, anti-Müllerian hormone, molecular marker, granulosa cells

## Abstract

AMH plays a critical role in ovarian follicular development and may influence reproductive performance. However, its genetic variation and association with litter size in goats remain unclear. In this study, three single-nucleotide polymorphisms (SNPs) in the *AMH* gene (g.89169447C>A, g.89169684G>A, and g.89170008T>C) were identified in Dazu Black goats. Association analysis showed that the g.89169447C>A locus was significantly associated with litter size at first parity (*p* < 0.05), while the g.89169684G>A locus was significantly associated with the average litter size across the first three parities (*p* < 0.05). Functional assays in granulosa cells indicated that the g.89169684G>A variant altered the expression of proliferation and apoptosis-related genes. These results suggest that the g.89169684G>A variant may serve as a potential molecular marker for improving reproductive performance in goats.

## Simple Summary

Reproductive efficiency is a key determinant of profitability in goat production, and litter size is one of its most important components. Identifying genetic markers associated with prolificacy can facilitate more efficient breeding strategies. Anti-Müllerian hormone (AMH) is known to play a central role in ovarian follicle development, yet its genetic variation in goats has been insufficiently explored. In this study, we identified polymorphisms in the *AMH* gene of Dazu Black goats and evaluated their association with litter size. One variant was significantly associated with litter size traits and showed measurable effects in ovarian granulosa cells. These findings provide preliminary evidence that *AMH* variation may contribute to litter size differences and could be considered in marker-assisted selection programs.

## 1. Introduction

Goats are widely distributed worldwide and possess abundant genetic resources, making them one of the most important livestock species for agricultural production. Litter size is a key indicator of reproductive performance in goats [1]. Therefore, the identification of genetic factors associated with prolificacy is of considerable importance for the development of marker-assisted selection strategies and the genetic improvement of reproductive traits [2]. The Dazu Black goat, a local breed originating from Dazu District, Chongqing, China, has been shaped through long-term natural and closed breeding over several centuries. This breed exhibits remarkable genetic stability and phenotypic consistency [3]. More importantly, it exhibits exceptional reproductive performance, with reported twin, triplet, and quadruplet birth rates of 47.27%, 40.02%, and 10.29%, respectively [4]. The average kidding rate of this breed can reach approximately 260% [5], substantially exceeding that reported for several other goat breeds, including Matou goats, Korean native goats, Boer goats, and improved Boer goats [6]. These characteristics make the Dazu Black goat an excellent model for investigating the genetic mechanisms underlying prolificacy in goats.

AMH is a glycoprotein member of the transforming growth factor-β (TGF-β) superfamily and plays a pivotal role in reproductive organ differentiation and ovarian follicular development [7]. The AMH gene spans more than 2.75 kb and contains five exons. It is located on chromosome 7 in goats, cattle, and horses and on chromosome 5 in sheep [8]. Increasing evidence suggests that AMH is closely associated with ovarian follicular reserve, fertility, reproductive lifespan, and superovulatory response in ruminants [9]. As a reproductive biomarker, circulating AMH concentrations have been shown to predict embryo production potential more accurately than either the presence of FecG mutations or preselection based on equine chorionic gonadotropin (eCG) responsiveness [10]. In female mammals, AMH is primarily secreted by granulosa cells of preantral and small antral follicles and acts in a paracrine manner. It inhibits the initial recruitment of primordial follicles and modulates follicular development by providing negative feedback from growing follicles, thereby preserving the primordial follicle pool and extending reproductive lifespan [11]. Following sexual maturation, AMH levels gradually decline with age due to the continuous depletion of the primordial follicle reserve [12]. Experimental studies have shown that exposure of neonatal mouse ovaries or human ovarian tissue to elevated AMH levels significantly reduces the number of growing follicles, thereby preventing premature depletion of ovarian reserves [13]. Furthermore, AMH suppresses follicle-stimulating hormone receptor (FSHR) expression in antral follicles, reducing follicular sensitivity to follicle-stimulating hormone (FSH) and consequently limiting follicular growth [14]. Therefore, AMH is widely regarded as a reliable indicator of ovarian reserve and reproductive potential [15]. With the advancement of molecular genetics, single-nucleotide polymorphisms (SNPs) in the AMH gene have been reported to be associated with reproductive performance in various mammalian species. For instance, De Conto et al. demonstrated that the AMH p.Ile49Ser polymorphism is associated with infertility in endometriosis [16]. In livestock species, a mutation (g.1996A>G) within the AMH gene has been reported to significantly affect egg-laying performance in Jinghai Yellow chickens [17]. In Holstein cattle, circulating AMH concentrations are associated with reproductive phenotypes, and the AMH SNP rs876084180 has been proposed as a potential predictor of reproductive performance [18]. In goats, studies investigating AMH genetic variation and its relationship with reproductive traits remain relatively limited. Previous research has identified an exon variant (g.89172108A>C) that may be associated with litter size at the second parity [19], suggesting a potential role of AMH polymorphisms in regulating reproductive performance. Moreover, the functional significance of naturally occurring coding-region variants in the caprine AMH gene remains largely unknown.

Single-nucleotide polymorphisms (SNPs) are important molecular markers that link genetic variation to phenotypic diversity and have been widely applied in goat breeding programs [20]. Previous studies have demonstrated significant associations between SNPs in fecundity-related genes and litter size traits. For example, SNPs located at nucleotide positions 735, 754, and 781 within exon 2 of the BMP15 gene were significantly associated with litter size in Black Bengal goats [21]. Similarly, a polymorphism (g.28317663A>C) in the INHA gene has been identified as a candidate marker for improving reproductive performance in Hainan Black goats [22]. Despite the recognized importance of AMH in ovarian physiology, studies investigating naturally occurring AMH variants in goats remain scarce, and the molecular mechanisms linking AMH genetic variation to reproductive performance have not been fully elucidated. In particular, the relationship between AMH genetic variation, granulosa cell function, and prolificacy has not been systematically investigated. Therefore, the objectives of the present study were to identify polymorphisms within the AMH gene, evaluate their associations with litter size traits in Dazu Black goats, and preliminarily explore the biological effects of a functional AMH variant in ovarian granulosa cells. These findings may provide new insights into the genetic regulation of prolificacy and facilitate the development of candidate molecular markers for goat breeding programs.

## 2. Materials and Methods

### 2.1. Animals

A total of 150 female Dazu Black goats with complete reproductive records (≥3 parities) were selected from Dazu Black Goat Conservation Farm, Chongqing Tengda Animal Husbandry Co., Ltd. (Chongqing, China), with permission from the farm owner. All animals were raised under similar management conditions. Blood samples were collected via jugular venipuncture and stored in EDTA-treated tubes for genomic DNA extraction. For granulosa cell isolation, ovarian tissues were collected from 15 healthy multiparous Dazu Black goats (1–3 years old) immediately after slaughter. A total of 30 ovaries were transported to the laboratory for subsequent granulosa cell isolation and culture. All animal procedures were conducted in accordance with the Guidelines for the Ethical Review of Laboratory Animal Welfare (GB/T 35892-2018) [23] and were approved by the Animal Ethics Committee of Southwest University (Approval No. IACUC-20220915-01).

### 2.2. Genomic DNA Extraction and SNP Identification

Genomic DNA was isolated from whole-blood samples using the phenol–chloroform extraction method. PCR amplification was performed in a 30 μL reaction volume containing 15 μL 2× Taq PCR Master Mix, 1 μL genomic DNA (~20 ng), 2 μL of each primer (5 pmol/μL), and 10 μL nuclease-free water. The thermal cycling program consisted of an initial denaturation at 95 °C for 5 min, followed by 35 cycles of 95 °C for 30 s, primer-specific annealing for 30 s, and 72 °C for 30 s, with a final extension at 72 °C for 5 min.

Amplified products were sequenced using an ABI 3730XL DNA Analyzer (Applied Biosystems, Foster City, CA, USA). Sequence chromatograms were inspected using Chromas software (version 2.6.6, Technelysium Pty Ltd., South Brisbane, Australia), and SNPs were identified by alignment with the AMH reference sequence (NC_030814.1). Only chromatograms with clear peak resolution and a Phred quality score ≥20 were included in the analysis. Primer sequences, amplicon sizes, and annealing temperatures are provided in Supplementary Table S1.

### 2.3. Granulosa Cell Isolation, Culture, and Transfection

Granulosa cells (GCs) were isolated from ovarian follicles (1–5 mm in diameter) according to a previously described method [24]. Ovaries were collected from cyclic adult goats during the breeding season, irrespective of the stage of the estrous cycle. Granulosa cell identity was previously verified by FSHR immunostaining using the same isolation protocol [25]. Cells were cultured in DMEM/F-12 medium supplemented with 10% fetal bovine serum and 100 IU/mL penicillin and 100 μg/mL streptomycin and maintained at 37 °C in a humidified atmosphere containing 5% CO_2_. Cells were transfected with AMH overexpression plasmids carrying either the A allele or the G allele at the g.89169684 locus, hereafter referred to as AMH-A and AMH-G, respectively, using Lipofectamine 3000 (Invitrogen, Carlsbad, CA, USA) according to the manufacturer’s instructions. Transfection efficiency was assessed indirectly by measuring AMH mRNA expression and protein secretion following plasmid transfection.

### 2.4. Enzyme-Linked Immunosorbent Assay (ELISA)

The concentration of AMH in culture supernatants was determined using a commercial goat AMH ELISA kit (Sinobestbio, Shanghai, China) following the manufacturer’s instructions. According to the manufacturer, the analytical sensitivity of the assay was 10 pg/mL, with intra-assay and inter-assay coefficients of variation below 8% and 10%, respectively. Each treatment group consisted of three independent biological replicates, and each sample was analyzed in five technical replicates. Absorbance was measured at 450 nm, and AMH concentrations were calculated using a standard curve generated from the supplied standards.

### 2.5. Gene and Protein Expression Analysis

To preliminarily evaluate the potential biological effects of the AMH variant in granulosa cells, genes associated with cell proliferation (PCNA), apoptosis (BAX, BCL2, and Caspase3), cell cycle regulation (CCND1, CCNE1, and CDK4), and reproductive signaling pathways (GDF9, SMAD4, FSHR, and LHR) were selected for expression analysis. At 24 h after transfection, total RNA was extracted from each treatment group using RNAiso Plus reagent (TaKaRa, Kyoto, Japan) and subsequently reverse-transcribed into first-strand cDNA according to the manufacturer’s instructions. Quantitative real-time PCR was performed using gene-specific primers (Table 1), and relative transcript abundance was calculated using the 2^−ΔΔCt^ method with β-actin serving as the reference gene. Primer specificity was verified by melting-curve analysis. For each treatment, three independent biological replicates were analyzed, and each biological replicate was measured using four technical replicates.

Protein extracts were prepared from treated granulosa cells using lysis buffer supplemented with protease inhibitors. Equal amounts of protein were separated by SDS–PAGE and subsequently transferred onto PVDF membranes. After blocking, membranes were incubated overnight at 4 °C with primary antibodies against PCNA (1:5000, Cat. No. 10205-2-AP, Proteintech, Wuhan, China), BAX (1:20,000, Cat. No. 50599-2-Ig, Proteintech), BCL2 (1:2000, Cat. No. 12789-1-AP, Proteintech), and β-actin (1:20,000, Cat. No. 66009-1-Ig, Proteintech). After washing, membranes were incubated with the corresponding HRP-conjugated secondary antibodies. Immunoreactive signals were detected using an enhanced chemiluminescence substrate and captured using a ChemiDoc imaging system (Bio-Rad, Hercules, CA, USA). Protein expression levels were normalized to β-actin, and band intensities were quantified using ImageJ software (version 1.53t, National Institutes of Health, Bethesda, MD, USA).

### 2.6. Statistical Analysis

Genetic diversity parameters, including allele frequencies, genotype frequencies, haplotypes, and polymorphism information content (PIC), were calculated using DnaSP and POPGENE 32 software. Observed homozygosity (Obs_Ho), observed heterozygosity (Obs_He), and effective allele number (Ne) were estimated using Microsatellite Toolkit. Hardy–Weinberg equilibrium (HWE) was assessed using the chi-square test. Association analysis between AMH gene polymorphisms and reproductive traits, including first-parity litter size (LS), average litter size across the first three parities (ALS3), first-parity average birth weight (ABW), and average birth weight across the first three parities (ABW3), was performed using the general linear model (GLM) procedure in SAS 9.4 (SAS Institute Inc., Cary, NC, USA). The statistical model was as follows:

*Y_ij_* = *μ* + *G_i_* + *e_ij_*

where *Y_ij_* is the observed phenotypic value, *μ* is the overall population mean, *G_i_* is the fixed effect of genotype, and *e_ij_* is the random residual error. Because reproductive traits were analyzed separately as first-parity traits and average traits across the first three parities, parity was inherently accounted for in the trait definition and was therefore not included as an additional effect in the model. Results are presented as mean ± standard deviation (SD), and multiple comparisons among genotypes were performed using the least significant difference (LSD) test. Statistical significance was declared at *p* < 0.05. All animals used for association analysis originated from the same conservation farm and were maintained under similar feeding and management conditions, thereby minimizing potential environmental variation among individuals.

For cell-based experiments, data are presented as the mean ± standard deviation (SD) from at least three independent biological replicates. Relative gene expression levels were calculated using the 2^−ΔΔCt^ method. Statistical differences among groups were evaluated using one-way analysis of variance (ANOVA) followed by the least significant difference (LSD) test. A value of *p* < 0.05 was considered statistically significant.

## 3. Results and Discussion

### 3.1. Identification and Genetic Characteristics of AMH SNPs

**Figure 1:**
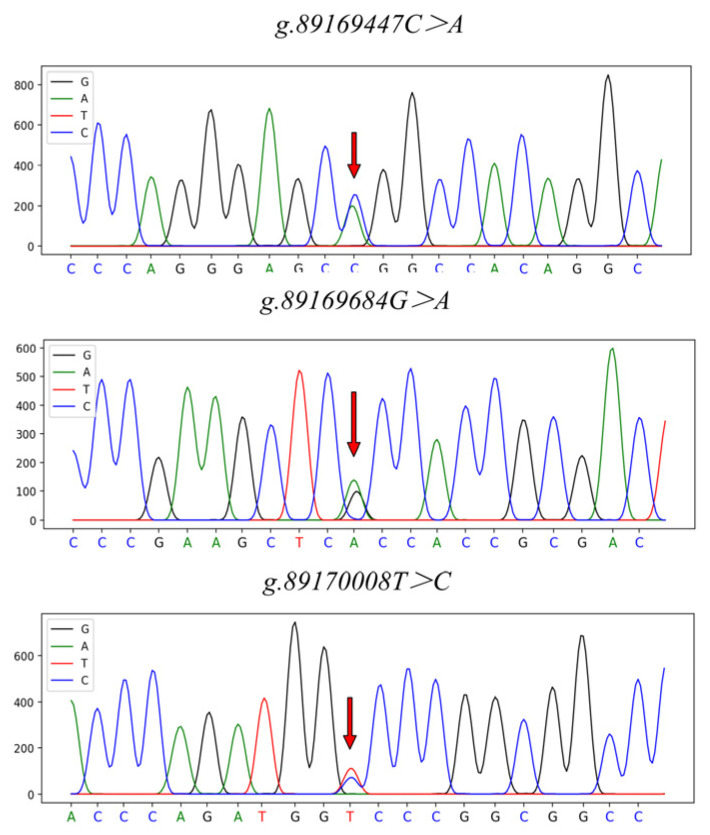
Sanger sequencing chromatogram of the AMH gene in DNA pools of Dazu Black goats.The red arrows indicate the corresponding SNP loci.

Three SNPs, namely g.89169447C>A, g.89169684G>A, and g.89170008T>C, were identified within the coding sequence (CDS) region of the AMH gene in Dazu Black goats through Sanger sequencing (Figure 1). Genotyping analysis (Table 2) revealed three genotypes (AA, AC, and CC) at the g.89169447C>A locus, represented by 77, 70, and 3 individuals, respectively. The AA genotype was the most frequent (51.33%), whereas the CC genotype occurred at a very low frequency (2.00%). At the g.89169684G>A locus, three genotypes (AA, AG, and GG) were identified, with 76, 65, and 9 individuals, respectively. The AA genotype was the predominant genotype (50.67%). At the g.89170008T>C locus, only two genotypes (CC and TC) were detected, represented by 131 and 19 individuals, respectively, with CC accounting for 87.33% of the population.

Population genetic analysis indicated that the g.89169447C>A and g.89169684G>A loci exhibited moderate polymorphism, whereas g.89170008T>C showed low polymorphism (Table 2). The g.89169684G>A and g.89170008T>C loci conformed to Hardy–Weinberg equilibrium, whereas the g.89169447C>A locus deviated from Hardy–Weinberg equilibrium. This deviation may be related to the extremely low frequency of the CC genotype and the limited number of individuals carrying this genotype.

The identification of polymorphic loci within the coding region of the AMH gene suggests that genetic variation in AMH may contribute to phenotypic variation in reproductive traits. However, some genotype groups were represented by relatively few animals, particularly the CC genotype at g.89169447C>A (*n* = 3) and the GG genotype at g.89169684G>A (*n* = 9). Such unbalanced genotype distributions may reduce statistical power and increase uncertainty in variance estimation, as phenotypic means can be disproportionately influenced by a small number of observations. In addition, multiple SNPs and reproductive traits were evaluated in the present study without independent population validation. Consequently, the possibility of false-positive associations cannot be completely excluded. Therefore, the observed associations should be interpreted cautiously and validated in larger and independent goat populations.

### 3.2. Association of AMH Polymorphisms with Litter Size Traits

Association analysis between AMH SNPs and reproductive traits (Table 3) revealed that, at the g.89169447C>A locus, goats carrying the AC genotype exhibited a significantly higher litter size at first parity than those carrying the AA or CC genotypes (*p* < 0.05). Specifically, the mean litter size of the AC genotype was approximately 64.5% higher than that of the AA genotype and 61.3% higher than that of the CC genotype. At the g.89169684G>A locus, goats with the AA and GG genotypes showed significantly higher average litter size across the first three parities than those with the AG genotype (*p* < 0.05), with increases of approximately 12.1% and 8.7%, respectively. No significant association was observed between the g.89170008T>C locus and litter size or birth weight (*p* > 0.05).

The magnitude of the observed genotype effects differed substantially between loci. At the g.89169447C>A locus, the AC genotype was associated with a markedly higher litter size at first parity compared with both homozygous genotypes, suggesting a potential heterozygote advantage. In contrast, the differences observed at the g.89169684G>A locus were more moderate, although they remained statistically significant for average litter size across the first three parities. These findings indicate that the biological impact of AMH polymorphisms on reproductive performance may vary depending on the specific mutation and reproductive trait evaluated.

AMH plays a critical role in ovarian follicle recruitment, activation, and development by regulating the transition of primordial follicles into the growing follicle pool and maintaining ovarian reserve [26]. Genetic variation in AMH has also been linked to reproductive traits, with previous studies reporting associations between AMH polymorphisms and hormone levels or fertility-related phenotypes [4,27]. Consistent with these findings, the present study identified two AMH polymorphisms, g.89169447C>A and g.89169684G>A, that were significantly associated with litter size traits in Dazu Black goats, further supporting the contribution of AMH genetic variation to reproductive performance. Similar associations have also been reported for several fecundity-related genes involved in follicular development and ovarian function, including GDF9, BMP15, BMPR1B, FSHR, and GLRB. Together with previous studies, the present findings further support the involvement of ovarian regulatory genes in the genetic control of reproductive performance. Accordingly, the AMH polymorphisms identified in this study may serve as potential candidate molecular markers associated with prolificacy in goats, although further validation in larger populations is required.

### 3.3. Effects of SNPs on mRNA Secondary Structure and Protein Coding

**Figure 2:**
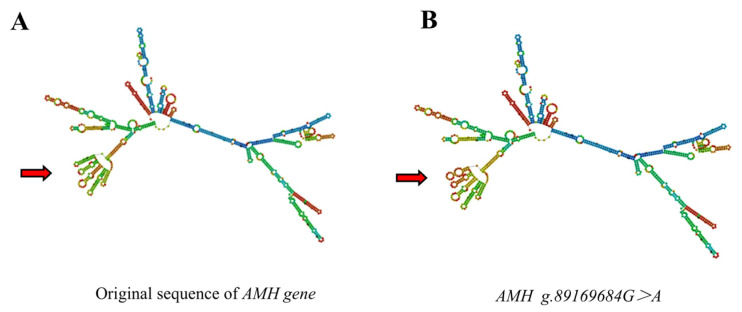
Predicted mRNA secondary structures of the AMH gene at the g.89169684 locus: (**A**) mRNA secondary structure carrying the A allele. (**B**) mRNA secondary structure carrying the G allele. The red arrows indicate the region corresponding to the g.89169684 locus. Colors were generated automatically by RNAfold to visualize the predicted RNA secondary structure and do not represent different experimental groups.

The RNAfold web server was used to predict the mRNA secondary structures of the AMH gene for allelic sequences at the g.89169447C>A and g.89169684 loci. No obvious structural change was observed between the allelic sequences at the g.89169447C>A locus. In contrast, the sequence carrying the G allele at g.89169684 showed alterations in predicted mRNA secondary structure compared with the sequence carrying the A allele (Figure 2A,B), accompanied by an increase in minimum free energy (MFE) from −411.2 kcal/mol for the A allele-containing sequence to −373.50 kcal/mol for the G allele-containing sequence. In addition, the free energy of the thermodynamic ensemble increased from −581.11 kcal/mol to −578.33 kcal/mol, suggesting reduced structural stability of the mutant transcript.

At the protein level, the g.89169447C>A variant was synonymous, whereas the allelic change at g.89169684 resulted in a missense variant causing an amino acid substitution from histidine to arginine. Previous studies have shown that non-synonymous substitutions may alter protein structure, stability, or biological activity, thereby affecting gene function [28,29]. Because histidine and arginine differ in their physicochemical properties, this substitution may influence local protein conformation or intermolecular interactions. However, these observations were based primarily on in silico predictions. Therefore, further functional analyses were performed in goat granulosa cells to evaluate the potential biological effects of this variant.

### 3.4. Functional Effects of the g.89169684G>A Variant in Granulosa Cells

**Figure 3:**
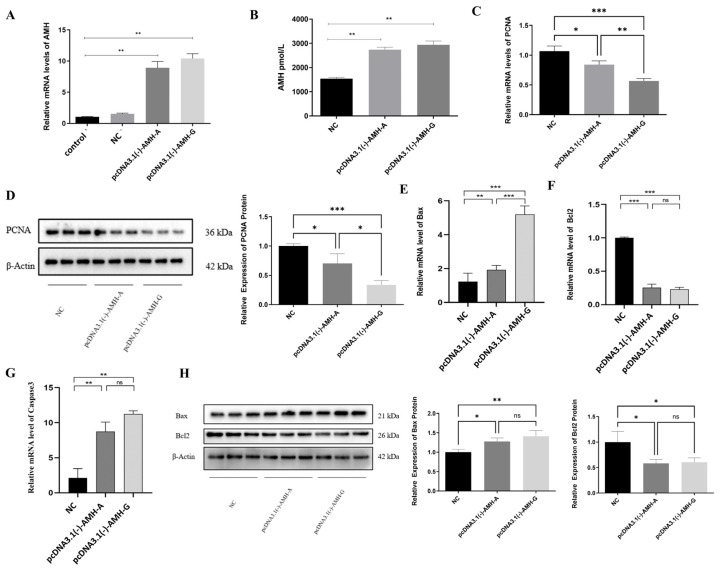
Functional effects of the g.89169684G>A variant in granulosa cells: (**A**) Relative AMH mRNA expression determined by RT-qPCR. (**B**) AMH protein concentration in granulosa cell culture supernatants measured by ELISA following plasmid transfection. (**C**,**D**) Relative mRNA and protein expression levels of PCNA. (**E**–**G**) Relative mRNA expression levels of Bax, Bcl2, and Caspase3. (**H**) Protein expression levels of Bax and Bcl2 determined by Western blot analysis. Note: Data are presented as mean ± SD from three independent biological replicates (n = 3). Relative mRNA expression levels were normalized to β-actin and calculated using the 2^−ΔΔCt^ method. Protein expression levels were normalized to β-actin. ns, not significant (*p* > 0.05); * *p* < 0.05; ** *p* < 0.01; *** *p* < 0.001.

Based on the predicted mRNA structural changes and amino acid substitution, the functional effects of the g.89169684G>A variant were further investigated in goat granulosa cells. AMH-A and AMH-G overexpression plasmids were constructed and transfected into granulosa cells. RT-qPCR and ELISA analyses confirmed that both constructs significantly increased AMH mRNA expression and protein secretion compared with the control group (Figure 3A,B), indicating successful overexpression.

Further analysis showed that both AMH-A and AMH-G significantly reduced the mRNA and protein expression levels of the proliferation marker PCNA, with a more pronounced effect observed in the AMH-G group (Figure 3C,D). In addition, both constructs significantly upregulated the expression of pro-apoptotic genes (Bax and Caspase3) and downregulated the anti-apoptotic gene Bcl2 (Figure 3E–H).

PCNA is widely recognized as a marker of cell proliferation [30], while members of the BCL2 family [31,32] and Caspase-3 [33] play central roles in the regulation of apoptosis. These expression changes suggest that the g.89169684G>A variant may influence pathways related to granulosa cell proliferation and apoptosis. AMH is generally considered a negative regulator of early follicular growth, partly by limiting granulosa cell responsiveness and restraining excessive follicle activation [11]. In this context, the reduced expression of PCNA observed after AMH-A and AMH-G overexpression is consistent with the inhibitory role of AMH in follicular growth. Notably, the AMH-G group showed a stronger reduction in PCNA expression than the AMH-A group, suggesting that the g.89169684G>A variant may enhance the inhibitory tendency of AMH-related signaling in granulosa cells. Meanwhile, the increased expression of Bax and Caspase3, together with decreased Bcl2 expression, indicates a shift in apoptosis-related molecular markers toward a pro-apoptotic profile. These changes may reflect an altered balance between granulosa cell survival and elimination, which is closely associated with follicular development and atresia. However, because the present study assessed marker gene and protein expression rather than direct proliferation or apoptosis assays, these findings should be interpreted as preliminary molecular evidence.

### 3.5. Potential Implications for Reproductive Regulation

**Figure 4:**
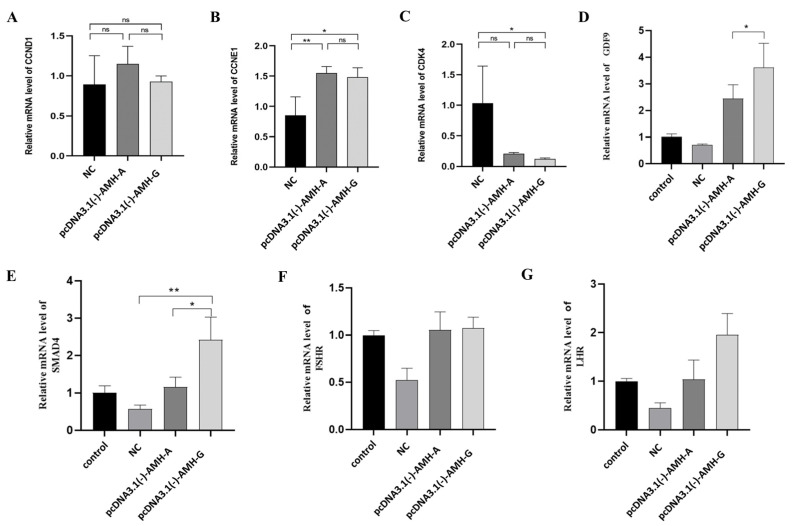
Potential implications of the g.89169684G>A variant for reproductive regulation: (**A**–**C**) Relative mRNA expression levels of CCND1, CCNE1, and CDK4 determined by RT-qPCR. (**D**–**G**) Relative mRNA expression levels of GDF9, SMAD4, FSHR, and LHR determined by RT-qPCR. Note: Data are presented as mean ± SD from three independent biological replicates (n = 3). Relative mRNA expression levels were normalized to β-actin and calculated using the 2^−ΔΔCt^ method. ns, not significant (*p* > 0.05); * *p* < 0.05; ** *p* < 0.01.

To further explore the potential regulatory effects of the g.89169684G>A variant, the expression levels of cell cycle-related genes (CCND1, CCNE1, and CDK4) were examined [34]. Neither AMH- A nor AMH- G affected CCND1 expression; however, both significantly upregulated CCNE1 and downregulated CDK4 expression (Figure 4A–C). CCNE1 [35] and CDK4 [36] are key regulators of cell cycle progression and play important roles in controlling granulosa cell proliferation and follicular development. The coordinated increase in CCNE1 and decrease in CDK4 expression observed in the present study suggests that the g.89169684G>A variant may alter the molecular regulatory network governing granulosa cell cycle progression. Although the precise biological significance of these changes remains to be clarified, the observed expression patterns are consistent with altered molecular regulation of granulosa cell proliferative activity.

Furthermore, the expression of genes associated with the TGF-β signaling pathway was evaluated. The AMH-Mut group exhibited significantly higher expression levels of GDF9 and SMAD4 than the AMH- A group (*p* < 0.05) (Figure 4D,E), whereas no significant differences were observed in FSHR or LHR expression (*p* > 0.05) (Figure 4F,G). GDF9 is an important oocyte-derived growth factor involved in follicular development and granulosa cell function [7], whereas SMAD4 serves as a central mediator of TGF-β family signaling, including AMH-related pathways [37,38]. The increased expression of GDF9 and SMAD4 in the AMH- G group may therefore indicate enhanced responsiveness of pathways associated with follicular regulation. In contrast, the absence of significant changes in FSHR and LHR expression suggests that the effects of the g.89169684G>A variant are more likely related to local intra-ovarian signaling networks than to gonadotropin receptor regulation.

Taken together, these findings suggest that the g.89169684G>A variant may influence reproductive regulation by modulating molecular pathways involved in granulosa cell function and TGF-β signaling. Notably, the g.89169684G>A locus represents a missense variant that causes a histidine-to-arginine substitution. As AMH belongs to the TGF-β superfamily, subtle alterations in protein structure may influence ligand–receptor interactions or downstream signaling efficiency. The differential expression of GDF9 and SMAD4 observed between the AMH- A and AMH- G groups provides preliminary evidence that this variant may affect AMH-related signaling pathways. However, the precise molecular mechanisms underlying these effects remain to be elucidated through future structural and functional studies.

## 4. Conclusions

In the present study, three SNPs were identified in the AMH gene of Dazu Black goats. Among them, the g.89169447C>A locus was significantly associated with litter size at first parity, while the g.89169684G>A locus was significantly associated with the average litter size across the first three parities. Functional analyses indicated that the g.89169684G>A variant may influence granulosa cell proliferation and apoptosis, as well as genes involved in reproductive regulation. Collectively, these findings suggest that the AMH g.89169684G>A locus represents a potential candidate molecular marker for litter size in Dazu Black goats.

## Figures and Tables

**Table 1:** PCR primer sequences.

Gene	Primer Sequence (5′ → 3′)	Product Size (bp)
*AMH*	F: GCTGCTTCACACGAAAGACC	148
	R: GCTCACGCATGAAGCACATT	
*GDF9*	F: GGTTGGACATCGGTATGGCT	126
	R: CAAAGGCATAGACAGGGGCG	
*FSHR*	F: ATGCGGTCGAACTGAGGTTT	94
	R: TTGGGAAGGTTCTGGAAGGC	
*LHR*	F: ATTCCGCCATCTTTGCTGAGAGTG	98
	R: AGCATCTGGTTCAGGAGCACATTG	
*SMAD4*	F: ACCCAAGACAGAGCATCAAGG	83
	R: GTCGGCAATAGGCATGGTGT	
*BAX*	F: CCAAGAAGCTGAGCGAGTGTCTG	88
	R: GTGTCCACGGCTGCGATCATC	
*β-actin*	F: TGATATTGCTGCGCTCGTGGT	186
	R: GTCAGGATGCCTCTCTTGCTC	
*BCL2*	F: TGTGGATGACCGAGTACCTGAACC	144
	R: GCCAGACTGAGCAGTGCCTTC	
*CDK4*	F: GCTGCTGCTGGAGATGCTGAC	100
	R: CTCTGCGTCACCTTCTGCCTTG	
*PCNA*	GTAGCCGTGTCATTGCGACTCC	140
	GCTCTGTAGGTTCACGCCACTTG	
*Caspase 3*	ATACCAGTTGAGGCAGAC	162
	TTAACCCGAGTAAGAATGT	
*CCND1*	TTCCTCTCCTATCACCGCCTGAC TCCTCTCTTCCTCCTCCTCCTC	170
*CCNE1*	AAGTGCTCCTGCCTCAGTATCCTC	124
	ATACAAGGCGGAAGCAGCAAGTAC	

**Table 2:** Genotype frequencies, allele frequencies, and genetic diversity parameters of AMH gene loci in Dazu Black goats.

SNPs	Genotype	Genotype Frequency, *n* (%)	Allele	Allelic Frequency (%)	Observed Heterozygosity	Observed Homozygosity	Ne	PIC	*p*
*g.89169447C* *>* *A*	AA	77 (51.33)	A	0.7467	0.4667	0.5333	1.61	0.31	0.004
	AC	70 (46.67)	C	0.2533					
	CC	3 (2.00)							
*g.89169684G* *>* *A*	AA	76 (50.67)	A	0.7233	0.4333	0.5667	1.67	0.32	0.31
	AG	65 (43.33)	G	0.2767					
	GG	9 (6.00)							
*g.89170008T* *>* *C*	CC	131 (87.33)	C	0.9367	0.1267	0.8733	1.13	0.11	0.41
	TC	19 (12.67)	T	0.0633					

Note: Ne represents the effective number of alleles, and PIC represents polymorphism information content. A PIC value < 0.25 indicates low polymorphism, 0.25–0.50 indicates moderate polymorphism, and >0.50 indicates high polymorphism. *p*-values were calculated using the chi-square (χ^2^) test. A *p*-value < 0.05 indicates deviation from the Hardy–Weinberg equilibrium (HWE).

**Table 3:** Association analysis between AMH gene polymorphisms and reproductive traits in Dazu Black goats.

SNPs	Genotype	Litter Size at First Parity	Average Litter Size Across First Three Parities	Average Birth Weight at First Parity (kg)	Average Birth Weight Across First Three Parities (kg)
*AMH* *g.89169447C* *>* *A*	AA	1.52 ± 0.51 ^b^	1.65 ± 0.19	2.26 ± 0.71	2.22 ± 0.39
	AC	2.50 ± 0.71 ^a^	2.17 ± 0.71	1.65 ± 0.5	1.55 ± 0.23
	CC	1.55 ± 0.52 ^b^	1.58 ± 0.45	2.08 ± 0.5	2.11 ± 0.36
*AMH* *g.89169684G* *>* *A*	AA	1.71 ± 0.56	1.67 ± 0.31 ^a^	2.05 ± 0.5	2.11 ± 0.39
	AG	1.40 ± 0.51	1.49 ± 0.28 ^b^	2.24 ± 0.73	2.12 ± 0.34
	GG	1.00 ± 0.53	1.62 ± 0.28 ^a^	3.50 ± 0.66	3.09 ± 0.35
*AMH* *g.89170008T* *>* *C*	CC	1.58 ± 0.55	1.64 ± 0.34	2.17 ± 0.63	2.14 ± 0.39
	TC	1.50 ± 0.71	1.50 ± 0.24	2.05 ± 1.64	2.03 ± 0.22

Note: Data are presented as mean ± SD. Different superscript letters (a, b) within the same column indicate significant differences among genotypes as determined by the least significant difference (LSD) test (*p* < 0.05).

## Data Availability

The datasets generated and/or analyzed during the current study are available from the corresponding authors upon reasonable request.

## Supplementary Materials

The following supporting information can be downloaded at: https://www.mdpi.com/article/10.3390/ani16121829/s1, Table S1: Primer sequence, product size, and annealing temperature of AMH in Dazu black goat.

## Abbreviations

The following abbreviations are used in this manuscript:

AMH	Anti-Müllerian hormone
SNPs	Single-nucleotide polymorphisms
GCs	Granulosa cells
RT–qPCR	Reverse transcription quantitative real-time PCR
ELISA	Enzyme-linked immunosorbent assay
PCNA	Proliferating cell nuclear antigen
BCL2	B-cell lymphoma 2
BAX	BCL2-associated X protein
CCND1	Cyclin D1
CCNE1	Cyclin E1
CDK4	Cyclin-dependent kinase 4
GDF9	Growth differentiation factor 9
SMAD4	SMAD family member 4
FSHR	Follicle-stimulating hormone receptor
LHR	Luteinizing hormone receptor
TGF-β	Transforming growth factor beta
MFE	Minimum free energy
HWE	Hardy–Weinberg equilibrium
PIC	Polymorphism information content
Ne	Effective number of alleles
Obs_Ho	Observed homozygosity
Obs_He	Observed heterozygosity
