# Supplementary material for: A Functional SNP in the AMH Gene Is Associated with Litter Size in Dazu Black Goats
Source: Animals (Basel). 2026 Jun 14;16(12):1829. doi: 10.3390/ani16121829 (PMC13296217; doi:10.3390/ani16121829)
Supplement: Supplementary file 1 [file animals-16-01829-s001.zip › animals-4311228-supplementary.pdf]

Supplementary Table S1. Primer sequence, product size and annealing temperature of AMH in Dazu black goat

Gene Name	Primer Name	Sequence (5′-3′)	Length (bp)	Temperature (°C)
AMH	AMH-1	F: CACACACTGTATCACTGCTC<br>R: ACCCCAAACAAGCAAAGATA	814	56
	AMH-2	F: CACCCTATCCATCACTTCTA<br>R: GAGTCCGCACCGAACAGCAG	818	63
	AMH-3	F: GCTGTCCTCAGACCCACTAC<br>R: GGGTCTTTCGTGTGAAGCAG	935	59
	AMH-4	F: CTGGCTGAACTCCCGTGTCT<br>R: CTTTATTGGGGCGATGACGG	1418	61
